# The diabetes cardiovascular outcomes trials and racial and ethnic minority enrollment: impact, barriers, and potential solutions

**DOI:** 10.3389/fpubh.2024.1412874

**Published:** 2024-10-25

**Authors:** Matthew R. Sinclair, Mariam Ardehali, Clarissa J. Diamantidis, Leonor Corsino

**Affiliations:** ^1^Department of Medicine, Division of Nephrology, Duke University School of Medicine, Durham, NC, United States; ^2^Duke Clinical Research Institute, Durham, NC, United States; ^3^Department of Medicine, Northwestern University Feinberg School of Medicine, Chicago, IL, United States; ^4^Department of Medicine, Wake Forest University School of Medicine, Winston-Salem, NC, United States; ^5^Department of Medicine, Division of Endocrinology, Metabolism, and Nutrition, Duke University School of Medicine, Durham, NC, United States; ^6^Duke Department of Population Health Sciences, Durham, NC, United States

**Keywords:** cardiovascular outcomes trials, minority enrollment, racial and ethnic minorities, type 2 diabetes, chronic kidney disease, cardiovascular disease

## Abstract

Type 2 diabetes (T2D) affects millions of individuals worldwide and is a well-documented risk factor for cardiovascular (CV) disease and chronic kidney disease, both of which are leading causes of mortality. Racial and ethnic minority groups in the US, including but not limited to Hispanic/Latino, non-Hispanic Black, and Southeast Asian individuals, are disproportionately burdened by both T2D and its adverse outcomes. In recent years, there have been numerous cardiovascular outcomes trials (CVOTs) on novel antidiabetic therapies, including the dipeptidyl peptidase-4 inhibitors, glucagon-like peptide-1 (GLP-1) receptor agonists (RAs), and sodium-glucose cotransporter-2 (SGLT2) inhibitors. CVOTs’s initial aim was to demonstrate the cardiovascular safety of these drugs. Unexpected CV and kidney protective effects were found, specifically among the GLP-1 RAs and the SGLT2 inhibitors. These benefits informed the new paradigm of the management of patients with T2D. However, some experts argued that the lack of racial and ethnic minority group representation in these trials represented a challenge. While the downstream effects of this lack of representation must be further elucidated, it is clear and recognized that efforts need to be made to include a more representative sample in future CVOTs, specifically including individuals from those groups most burdened by T2D and its complications, if clinicians are to have an accurate picture of the benefits and potential pitfalls of utilizing these drugs in a real-world setting. In this comprehensive review, we briefly summarize the significant findings from the CVOTs, report the lack of representation of Hispanic/Latino, non-Hispanic Black, and Southeast Asian individuals in the CVOTs, investigate the barriers to recruiting racial and ethnic minority groups into clinical trials, and suggest potential solutions to overcome these obstacles at the patient-, provider-, and sponsor/system-level in future trials.

## Introduction

Diabetes mellitus affects over 422 million individuals worldwide, the majority of whom suffer from type 2 diabetes (1). Type 2 diabetes (T2D) is a well-documented risk factor for cardiovascular disease (CVD), chronic kidney disease (CKD), and death (2, 3). While T2D is an epidemic, compared to non-Hispanic White individuals, racial and ethnic minority individuals are disproportionately burdened by T2D (4–7). The individuals who constitute racial and ethnic minority populations differ throughout the world. In the United States (US), Hispanics/Latinos and non-Hispanic Black individuals represent the largest proportion of the racial and ethnic minority groups. By 2060, it is predicted that Hispanics/Latinos will make up nearly 1/3 of the US population, and Black individuals will comprise 15% of the US population (8). Hispanic/Latino individuals are 17% more likely and non-Hispanic Black individuals are 60% more likely to have T2D than non-Hispanic White individuals, respectively, with more than 50% of US Hispanics/Latinos and non-Hispanic Black individuals estimated to develop T2D during their lifetime (4, 5). Further, Asians in the US are quickly growing and are also disproportionately burdened by T2D. Southeast Asians in particular (i.e., India, Pakistan, Bangladesh, Nepal, Sri Lanka, Bhutan, or the Maldives) have been found to be 3.4 times more likely to develop T2D compared to non-Hispanic White individuals (9).

Racial and ethnic minority individuals with T2D are more likely to suffer complications than non-Hispanic White individuals with T2D (7, 10). The many reasons for these disparities in risk factors and outcomes are complex, extend beyond biological differences, and often include modifiable socioeconomic, neighborhood, psychosocial, and behavioral factors (6). While addressing all of these factors is critical to attenuate healthcare inequities, there is also a critical need to identify therapeutic targets to mitigate the disproportionate risk of adverse outcomes among racial and ethnic minority individuals with T2D. Therefore, properly representing these groups in clinical trials and implementing evidence-based therapies in the real-world setting to improve patient outcomes is vital.

During the 1990s, partially in response to the growing global burden of T2D, several new agents for treating diabetes, such as thiazolidinediones, came onto the market. Many of these drugs were approved solely on studies that showed improvements in glycemic control, without evaluation for safety. However, post-marketing data indicated the potential for cardiovascular (CV) harm (11). Based on this evidence, the US Food and Drug Administration (FDA) recommended in 2008 to establish the CV safety of new antidiabetic therapies before their market approval. This led to multiple CV outcomes trials (CVOTs). Most notably for the dipeptidyl peptidase-4 (DPP-4) inhibitors, glucagon-like peptide-1 (GLP-1) receptor agonists (RAs), and sodium-glucose cotransporter-2 (SGLT2) inhibitors (11, 12). Although the goal of the CVOTs was primarily to establish CV safety, unexpected benefits of these drugs emerged. The CV and kidney protective effects of the GLP-1 RAs and the SGLT2 inhibitors in particular has significantly changed the management of patients with T2D, who are at increased risk for adverse CV and kidney outcomes (13–16).

While the study results of various CVOTs has changed the management of T2D, racial and ethnic minority individuals have been underrepresented in most studies. As T2D and its complications disproportionately burden racial and ethnic minority individuals, the impact of underrepresentation of these groups must be better elucidated. The overarching purpose of this manuscript is to briefly summarize the results of currently published CVOTs (including trials with a primary composite kidney endpoint), review minority representation in CVOTs, and examine the impact, barriers, and potential solutions for this underrepresentation. Lastly, we discuss strategies to increase recruitment of racial and ethnic minority individuals in future CVOTs.

### Summary of CVOTs results

#### Selection of CVOTs

The selection of clinical trials to be included in this manuscript began with an initial literature search for CVOTs and diabetes using PUBMED and Google Scholars as search engines. The search was directed by the investigators and a team of librarians. Trials identified included those found in *“The American Diabetes Association Cardiovascular Disease and Risk Management: Standards of Medical Care in Diabetes—2020.”* (17) Furthermore, the investigators also included trials that that were ongoing or published after 2020, presented at the national or international level, and deemed to be clinically impactful. Studies that were not clinical trials and/or did not specifically examine cardiovascular and/or kidney outcomes as primary endpoints were excluded.

#### Cardiovascular outcomes

The DPP-4 inhibitors were the first of the newer classes of T2D medications to undergo trials addressing CV safety. While none of the DPP-4 inhibitor trials demonstrated CV benefit, both Saxagliptin and Alogliptin trials suggested an increased risk of hospitalizations for heart failure. The other medications in the class did not demonstrate the same adverse events (14, 18–23). Most long-acting, injectable GLP-1 RAs have reduced 3- or 4-point major adverse cardiac events (MACE) in the CVOTs. However, while oral semaglutide and continuous subcutaneous infusion of exenatide have shown CV safety, they have not demonstrated the same CV benefit as the long-acting formulations (24–31). It is unclear whether differences in mechanisms of actions of these particular formulations versus factors related to these specific trials contributed to the lack of significant CV benefit. Most SGLT2 inhibitors have demonstrated significant CV benefit and reduction in MACE in patients with T2D, with and without heart failure (32–39). Ertugliflozin is the only SGLT2i that did not specifically demonstrate CV benefit, although it was non-inferior in terms of safety (40).

#### Renal outcomes

While the DPP-4 inhibitor trials focused primarily on CV outcomes, a few GLP-1 RA trials did examine key secondary renal outcomes. The “Cardiovascular and Renal Outcomes with Efpeglenatide in Type 2 Diabetes” (AMPLITUDE-O) trial (in which nearly 1/3 of patients had CKD), noted a reduction in a composite renal outcome (41). In contrast, a recently published pool analysis of two other CVOTs noted a significant lowering in albuminuria, slowing of eGFR slope decline, and time to persistent eGFR reduction (42). The trial “A Research Study to See How Semaglutide Works Compared to Placebo in People with Type 2 Diabetes and Chronic Kidney Disease” (FLOW) is the first GLP-1 RA trial to primarily examine renal outcomes in participants with CKD and T2D. While the trial was anticipated to be completed in 2024, encouraging results informed early trial termination as recommended by the DSMB (43, 44). The full results were just published in May 2024 and showed a 24% relative risk reduction in the composite of major kidney disease events and CV death (45). Multiple trials examined primary renal endpoints for SGLT2 inhibitors in patients with and without CKD and T2D, and demonstrated a reduction in the progression of kidney disease, as well as markers of CKD. Furthermore, the “Empagliflozin in Patients with Chronic Kidney Disease” (EMPA-KIDNEY) trial demonstrated safety and effectiveness in patients with advanced CKD (13, 46–48). See Tables 1–3 for more information about the CVOTs.

**Table 1:** Summary of dipeptidyl peptidase-4 inhibitor cardiovascular outcomes trials.

Trial	Year trial published	Drug	Major CV/renal benefit(s)	Notable adverse events
EXAMINE (23)	2013	Alogliptin	None	None
SAVOR-TIMI 53 (22)	2013	Saxagliptin	None	Increased HF hospitalizations
TECOS (19)	2015	Sitagliptin	None	None
CARMELINA (21)	2019	Linagliptin	None	None
CAROLINA (20)	2019	Linagliptin	None	None

**Table 2:** Summary of glucagon-like peptide-1 receptor agonist cardiovascular outcomes trials.

Trial	Year trial published	Drug	Major CV/renal benefit(s)	Notable adverse events
ELIXA (30)	2015	Lixisenatide	None	None
LEADER (29)	2016	Liraglutide	3P^*^-MACE^†^	None
SUSTAIN-6 (28)	2016	Semaglutide	3P^*^-MACE^†^	None
EXSCEL (26)	2017	Exenatide	None	None
HARMONY OUTCOMES (25)	2018	Albiglutide	3P^*^-MACE^†^	None
REWIND (24)	2019	Dulaglutide	3P^*^-MACE^†^	None
PIONEER-6 (27)	2019	Semaglutide	None	None
AMPLITUDE-O (41)	2021	Efpeglenatide	4P^*^-MACE^†^/2P^*^-Renal Outcomes	None
FREEDOM-CVO (31)	2021	Exenatide	N/A	None
FLOW (45)	2024	Semaglutide	3P*-Renal Outcomes & CV Death	None

* *P=Point; †MACE = Reduction in Major Adverse Cardiac Events; CV=Cardiovascular.*

**Table 3:** Summary of sodium-glucose cotransporter-2 inhibitor cardiovascular outcomes trials.

Trial	Year trial published	Drug	Major CV/renal benefit(s)	Notable adverse events
EMPA-REG OUTCOME (39)	2015	Empagliflozin	3P^*^-MACE^†^/CV Death	Genital Infection
CANVAS (35)	2017	Canagliflozin	3P^*^-MACE^†^	Amputation
CANVAS-R (103)‡	2017	Canagliflozin	3P^*^-Renal Outcomes	Amputation
DECLARE-TIMI 58 (38)	2018	Dapagliflozin	↓ risk of HF hospitalization/CV death	DKA and Genital Infections
DAPA-HF (34)	2019	Dapagliflozin	↓ risk of worsening HF/death from CV causes	None
CREDENCE (48)	2019	Canagliflozin	↓ risk of HF hospitalization/CV death	None
VERTIS-CV (40)	2020	Ertugliflozin	None	None
EMPEROR-REDUCED (36)	2020	Empagliflozin	↓ risk of HF hospitalization and serious renal outcomes	Uncomplicated Genital Infection
DAPA-CKD (47)	2020	Dapagliflozin	↓ risk of death from renal causes, ↓ risk of HF hospitalization/CV death	None
SCORED (33)	2020	Sotagliflozin	↓ risk of HF hospitalization/CV death	Diarrhea, Genital Mycotic Infections, Volume Depletion, DKA
SOLOIST-WHF (61)	2020	Sotagliflozin	↓ risk of HF hospitalization/CV death	Hypoglycemia
EMPEROR-PRESERVED (32)	2021	Empagliflozin	↓ risk of HF hospitalization	Genital Infection/UTI/hypotension
DELIVER (37)	2022	Dapagliflozin	↓ risk of HF events/CV death	None
EMPA-KIDNEY (46)	2022	Empagliflozin	↓ risk of hospitalization from any cause	None

^*^P=Point; ^†^MACE = Reduction in Major Adverse Cardiac Events; ‡CANVAS-Renal(R) was designed as a second CANVAS-like, double-blind, randomized, placebo-controlled trial to be analyzed jointly with CANVAS. The linked publication reports the results of a prespecified, exploratory analysis of the long-term effects of canagliflozin on primary renal outcomes.

### The impact of CVOTs on T2D management

Numerous multinational, randomized CVOTs have revolutionized how we treat patients with T2D. This overwhelming evidence has informed the new recommendations from American Diabetes Association (ADA), European Association for the Study of Diabetes (EASD), American College of Cardiology (ACC), American Heart Association (AHA), and Kidney Disease: Improving Global Outcomes (KDIGO), to name a few (49–52). However, a topic of ongoing debate is the lack of representation of racial and ethnic minority populations in these trials. This is significant as these groups are disproportionally affected by T2D and its complications.

### Racial and ethnic minority enrollment in the CVOTs

#### Race/ethnicity in the US vs. globally

To fully comprehend the importance of racial and ethnic minority enrollment in the CVOTs and its implications for the generalizability and application of the results, it is necessary to understand what the concepts of race and ethnicity mean for the US vs. globally. The importance of race and ethnicity and its impact is very complex and beyond the scope of this review. However, we should briefly acknowledge that race and ethnicity are social constructs with different meanings worldwide (53). The US, compared to other countries included in multinational CVOTs, is a country made up mostly of immigrants. Despite slavery being outlawed for over 150 years in the US, its legacy continues to impact Black communities. Conversely, while other nations around the globe have their own histories of race relations, they are unique and different from those in the US.

Further, when we start discussing ethnicity in the US compared to the rest of the world, things become convoluted. Specifically, when we talk about Hispanics/Latinos, this terminology is mostly applied in the US. It is an umbrella term that refers primarily to individuals who trace their origins to countries conquered by Spain or that speak a language originating from Latin (54). While individuals who meet these criteria might share a common language and even some cultural similarities, they represent a heterogeneous group. When looking globally, however, the terms Hispanic/Latino are often not even recognized. Because some of the CVOTs recruited from Latin American and European countries, this may explain why Hispanic/Latino ethnicity was often not reported.

The term Asian is recognized globally as individuals from or with heritage background in the Asian continent. In the US, Asian refers to “A person having origins in any of the original peoples of the Far East, Southeast Asia, or the Indian subcontinent including Cambodia, China, India, Japan, Korea, Malaysia, Pakistan, the Philippine Islands, Thailand, and Vietnam” (55). Similar to Hispanics/Latinos, Asians are a heterogenous group. In the CVOTs, Asians are reported primarily as a singular group and individual Asian subgroups were usually not reported. This is an important limitation as certain Asian subgroups (i.e. Southeast Asians) have a higher reported prevalence of T2D, compared to other Asian subgroups (9, 56).

When we discuss increasing study participation for racial and ethnic minority individuals in global studies, we must acknowledge that increasing the percentage of Black, Hispanic/Latino, or Asian participants in the US is not always comparable to increasing the percentage of Black, Hispanic/Latino, or Asian participants in Europe, Latin America, Africa, Asia, or other parts of the world. Furthermore, reporting one “overall percentage” number is not sufficient. Rather, it is critical to note the percentage of racial and ethnic minorities enrolled in the trial from the US compared to the rest of the world. When examining the published CVOTs, there is no delineation of sracial and ethnic minority individuals by those enrolled in the US vs. other parts of the world, making it challenging to fully extrapolate the significance of the findings as they relate specifically to the health of racial and ethnic minority individuals.

Lastly, it is important to understand that race is a social construct, often with minimal biological significance. Therefore, we should never explain observed differences in a trial to race alone. If trialists want to use ancestry for things like “precision medicine,” (57, 58) which focuses on biological differences between individuals and how that may cause clinically meaningful pharmacogenomic and pharmacokinetic differences, the onus falls on them to explain the biological plausibility of such differences, moving beyond a race-only based approach. When considering any differences that arise in a treatment effect that may appear to separate on racial or ethnic lines, we must consider all other factors (i.e., social, economic, cultural, etc.) that could contribute to those findings. For the purposes of this review article, we focus on underrepresentation of racial and ethnic minorities in CVOTs recognizing that is not the only solution to ameliorate health disparities and advance towards health care equity. Due to difficulties of generalizing racial and ethnic minority classifications globally, we express a US-centric focus toward representation and future recruitment of specifically Black, Hispanic/Latino, and Asian individuals in the CVOTs. However, many of the challenges and strategies are not unique to the US or to CVOTs. They may be extrapolated to other nations, with the caveat of needing to fully understand race relations and their effect on health outcomes in those countries.

#### Black participants’ representation

**Figure 1:**
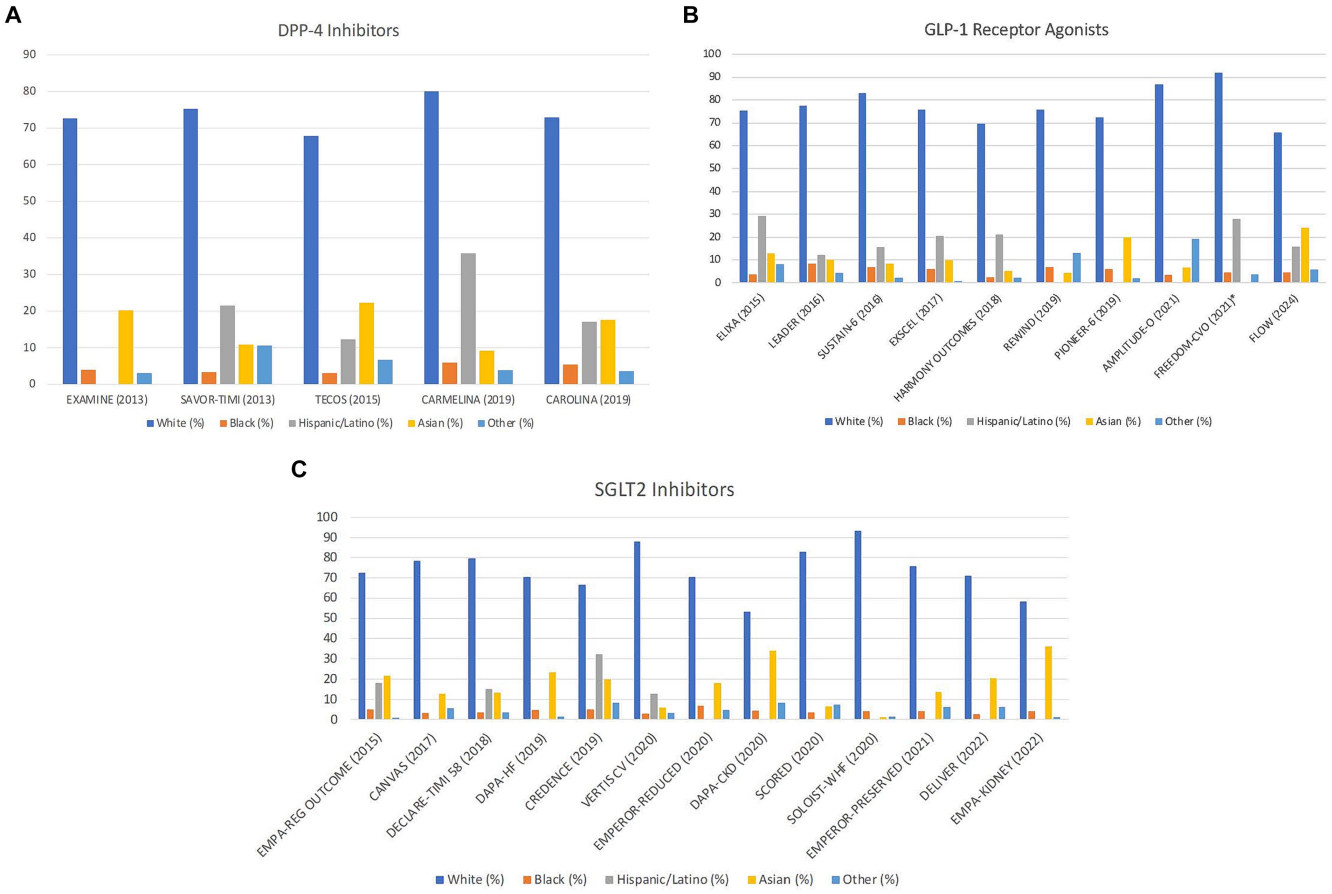
Racial and ethnic breakdown of cardiovascular outcomes trials by publication date and drug class. **(A)** Distribution of DPP-4 inhibitor trials by race/ethnicity with publication date. Missing bars indicate unreported data. “Other” includes any patient that does not fit into these categories. **(B)** Distribution of GLP-1 agonist trials by race/ethnicity with publication date. Missing bars indicate unreported data. “Other” includes any patient that does not fit into these categories. *FREEDOM-CVO did not include data for Asians, so this population may be included in the “other” section. **(C)** Distribution of SGLT2 inhibitor trials by race/ethnicity with publication date. Missing bars indicate unreported data. “Other” includes any patient that does not fit into these categories.

Despite disproportionately suffering the burden of T2D and its complications, specifically in the US, a low proportion of Black individuals were enrolled in the CVOTs. While Black individuals comprise 13.6% of the overall US population, Black participants ranged from 2.3 to 9.7%. In DPP-4 inhibitor trials published between 2013 to 2019, the trial with the highest representation of Black participants was the “Cardiovascular and Renal Microvascular Outcome Study With Linagliptin (CARMELINA)” with 5.8% (21). The trial with the lowest reported number of Black participants was the “Trial Evaluating Cardiovascular Outcomes with Sitagliptin (TECOS),” with 3% (19) (Figure 1A). In the CVOTs evaluating the GLP-1 RAs, published between 2015 to 2024, Black participant representation was the highest for AMPLITUDE-O at 9.7% (41). The lowest representation of Black individuals in GLP-1 RA trials was 2.3% in “Albiglutide and Cardiovascular Outcomes in Patients with Type 2 Diabetes and Cardiovascular Disease (HARMONY OUTCOMES)” (25) (Figure 1B). Unsurprisingly, CVOTs looking at SGLT2 inhibitors published between 2015 to 2022 also had a very low representation of Black participants. The trial with the highest representation at 6.8% was “Cardiovascular and Renal Outcomes with Empagliflozin in Heart Failure EMPEROR-REDUCED” (36). The trial with the lowest representation was “Dapagliflozin in Heart Failure with Mildly Reduced or Preserved Ejection Fraction (DELIVER) at 2.5% (37) (Figure 1C).

#### Hispanic/Latino participants’ representation

The representation of Hispanics/Latinos in the CVOTs, which currently represents 18.9% of the US population (59), was variable, ranging from 7.7% up to 32.3% (13, 15, 16, 59, 60). Of note, it is important to highlight that 15 trials did not report Hispanic/Latino representation, the majority being SGLT2 inhibitor CVOTs (24, 27, 32–37, 41, 46–48, 61). For trials looking at DPP-4 Inhibitors and reporting Hispanic/Latino ethnicity, representation was highest in “Saxagliptin and Cardiovascular Outcomes in Patients with Type 2 Diabetes Mellitus (SAVOR-TIMI 53)” at 21.4% and lowest in TECOS with 12.3% (19, 22) (Figure 1A). In GLP-1 RA trials, Hispanic/Latino representation was highest in “Lixisenatide in Patients with Type 2 Diabetes and Acute Coronary Syndrome (ELIXA)” at 29.1% and lowest in “Effects of Once-Weekly Exenatide on Cardiovascular Outcomes in Type 2 Diabetes (EXSCEL)” at 7.6% (26, 30) (Figure 1B). In SGLT2 inhibitor CVOTs, the highest representation was in “Canagliflozin and Renal Outcomes in Type 2 Diabetes and Nephropathy (CREDENCE)” at 32.3%, whereas the lowest representation was in “Cardiovascular Outcomes with Ertugliflozin in Type 2 Diabetes (VERTIS CV)” at 12.6% (40, 48) (Figure 1C).

#### Asian participants’ representation

Asians constitute approximately 7% of the US population (62). Asian representation in the CVOTs was variable, ranging from 1.2 to 36.2%. In DPP-4 Inhibitor trials, Asian representation was highest in TECOS at 22.3% and lowest in CARMELINA at 9.2% (19, 21) (Figure 1A). For GLP-1 RA trials, percentage of Asians was highest in “Oral Semaglutide and Cardiovascular Outcomes in Patients with Type 2 Diabetes” (PIONEER-6) at 19.8% and lowest in “Dulaglutide and Cardiovascular Outcomes in Type 2 Diabetes” (REWIND) at 4.4% (24, 27). Notably, despite recruiting in Asia, the “Subcutaneous Infusion of Exenatide and Cardiovascular Outcomes in Type 2 Diabetes” (FREEDOM-CVO) did not report percentage of Asian participants (31) (Figure 1B). Among the SGLT2 inhibitor trials, Asian inclusion was highest in EMPA-KIDNEY at 36.2% and lowest in “Sotagliflozin in Patients with Diabetes and Recent Worsening Heart Failure” (SOLOIST-WHF) at 1.2% (46, 61) (Figure 1C).

#### Other participants’ representation

It is important to note that there are other racial subgroups, including American Indians/Alaska Natives and Native Hawaiians or Other Pacific Islanders, who also experience disproportionately high rates of T2D and its complications (63). In the majority of CVOTs, individuals from these groups were included in the “Other” category. Because these individuals make up a relatively small percentage of the US population (~1.6%) and an even smaller percentage outside of the US, we did not specifically focus on them in this review paper that examined multinational clinical trials (64). However, given the disparities these groups face, it is important that their recruitment be considered in future clinical trials.

## Discussion

### Barriers and potential solutions to improve minority representation in clinical trials

Disparities exist in recruiting racial and ethnic minority individuals to clinical trials in general (65–67). The CVOTs demonstrate this same trend. This is a significant issue as racial and ethnic minority individuals stand to glean the most benefit from trial inclusion, since they are disproportionately burdened by T2D and renal and CV complications related to their diabetes.

Furthermore, researchers must strive to recruit a representative sample of the population in their trials to accurately measure the effects of these therapies in a real-world setting and in the diverse population. If individuals from racial and ethnic minority groups continue to be underrepresented in medication trials, questions will continue to persist regarding how well research findings can be generalized and whether the response and the reporting of adverse effects accurately represents the populations that will be utilizing the medications (68). Accomplishing equity in trials will require specifically implementing strategies to minimize well-documented barriers to recruit a representative number of individuals. These barriers can be divided into three categories: (1) Patient-Level, (2) Provider-Level, and (3) Sponsor/System-Level (69). While there exists overlap between categories, structuring barriers in this manner allows for a comprehensive understanding of where the problems exist and a nuanced conversation of potential strategies to address some of these barriers. While the many barriers are too numerous to fully elucidate, we will discuss some of the most salient barriers to recruiting racial and ethnic minority individuals to clinical trials and offer some solutions to consider when designing future studies.

### Patient-level barriers

On the patient-level, time commitment, lack of perceived benefit, inadequate compensation, worries around confidentiality, and mistrust are the most cited barriers (69–72, 74–76, 79, 82). The follow-up visits and paperwork required for many clinical trials can be daunting. Furthermore, this barrier is often coupled with a lack of interest in participating in the trial in the first place, as benefits from the therapies still need to be fully understood (70–72). Lack of understanding of a trial’s possible benefits could be partly due to lower health literacy (73). This lack of comprehension of potential trial benefits not only contributes to poor recruitment, but also builds on the fear that by participating, participants may be exposing themselves to harm and/or exploitation, which could then lead to further distrust between physicians/researchers and minoritized communities (70–72). There is also evidence that racial and ethnic minority individuals feel inadequately compensated (70). Patients are also reluctant to participate in studies out of fear of losing confidentiality and privacy (74–76). Despite the process of informed consent, deep-rooted distrust of researchers among racial and ethnic minority individuals persist, as well as a fear of what will happen to their personal information (68). Mistrust of the US research enterprise and medical system are due to many factors, including feelings of discrimination and feeling like they are not being taken seriously by the medical community (77–80). These fears stem from past unethical medical research practices, negative experiences by community members, and the relationship or reputation between the research institution and the community (68, 81).

### Provider-level barriers

Physicians, non-physician providers, and researchers contribute to the lack of representation in clinical trials. Providers are often not aware of ongoing clinical trials that may benefit their patient population. Even once they are aware of active clinical trials, providers may make biased assumptions about racial and ethnic minority patients, lack of suitability, and concerns regarding long-term survival, and trial comprehension. For example, the time and effort it takes to use an interpreter may discourage physicians and researchers from approaching non-English speaking patients about studies they may qualify for. Additionally, a provider may assume that a patient who has missed multiple appointments would not be the best candidate to follow and adhere to the requirements of a clinical trial. Communication barriers also play a role. If providers are unable to articulate the potential benefits of a trial in a way the patient can understand while also being able to answer relevant questions about the trial, it is unlikely that they will introduce the patients to research opportunities (69, 70, 74, 82–84). Also, the increasing number of expectations and burnout experienced by providers limits their time and willingness to introduce research participation to their patients (85). All of these factors contribute to the withdrawal of potential trial participants from consideration, often before attempting to recruit them in the first place.

### Sponsor/system-level barriers

Sponsor/system-level barriers include lack of transportation, which precludes the inclusion of potential participants with an inability to reach the health care facilities or research facilities (69, 70, 74, 76, 82). Previous studies have shown that Hispanic/Latino patients tend to have less access to transportation compared to non-Hispanic White individuals and often cite it as a barrier to care (86). Another sponsor/system-level is the lack of representation of racial and ethnic minority physicians, research staff, and investigators. Recent data shows that although minoritized individuals represent about 35% of the general US population, only 13% of physicians are under-represented minoritized (URM) individuals (87). The percentage of URM scientists in the US is not much higher at ~16% (88). There is evidence that homophily, which is the tendency to form stronger connections with individuals who share one’s defining characteristics, between providers and patients, may lead to improved uptake in health behaviors (89, 90). Unfortunately, since there is such a paucity of URM individuals in medicine, homophily between providers and potential minoritized research participants is severely lacking. A final pertinent and often cited barrier posed by physicians and research staff is the lack of culturally appropriate information in the native language of their patients and potential research participants (69, 82, 90). Language poses a significant barrier to non-native English speakers, as information regarding participation in the trial may not be fully or adequately understood by the patient. Additionally, patients are less likely to participate in studies that are not compatible with their religious and cultural practices. Furthermore, since clinical trials often recruit from clinics, lack of access to healthcare among certain racial and ethnic groups leads to less representation in the clinic and less access to trial participation (69, 82, 90). It is also important to note that systemic and structural racism, which often makes the healthcare system and associated resources less accessible to communities of color and other marginalized groups, is a major underlying factor of many of these sponsor/system-level (91).

### Patient-level solutions

To overcome the obstacles to recruiting racial and ethnic minority individuals into clinical trials, strategies must be developed and implemented to address barriers. Challenges and potential solutions are summarized in Table 4. On the patient-level, providing the patient with an adequate opportunity to gather information about the trial, whether that be through conversations with the research staff or through a question and answer session, has been shown to increase study recruitment (70). Fears of confidentiality may be lessened by an honest and upfront description of the research project with patients, offering a clear explanation of what will be done with their private health information. The researchers should share results with the participant(s). In terms of lack of interest and worries about time commitment, one possible solution is offering patient incentives such as monetary compensation, which could provide a tangible reward for patients for participation and improve recruitment rates (92, 93). However, as noted by *Occa* et al. *“offering money and increased compensation for research participation is also problematic, especially while recruiting patients from low SES and minority groups due to the potential for coercion.”* Further, it may increase suspicion that the researcher is not acting in the best interest of the research participant (90). Therefore, incorporating the community’s views and perspectives while designing a trial and determining the level and type of compensation for study participation is also of value. One possible solution to address the mistrust in the medical system and research involves partnering with community members, physicians, leaders, and organizations to encourage active participation from the community in formulating the research questions, study design and recruitment strategies (69, 75, 76, 92–95). By working collaboratively with the community, including trusted leaders and community members, investigators are able to introduce clinical trials through trusted, respected individuals within the community. For example, leaders of religious organizations can communicate information about valuable clinical trials to parishioners. By partnering with community organizations, recruitment is expanded outside of the clinic, offering access to trial participation to those with minimal healthcare access. Additionally, it is important that we acknowledge the past negative interactions communities have had with the medical establishment, and the ongoing challenges those communities face when it comes to trusting clinicians and researchers due to previous unethical practices. Full transparency will allow investigators to demonstrate trustworthiness, which will increase participants’ confidence in participating in research studies (68).

**Table 4:** Present problems and potential solutions to improve recruitment of racial and ethnic minorities to research trials.

Present problems	Potential solutions
Worries around confidentiality/mistrust of medical system	Adequate opportunity to gather information (open conversation, Q + A) Partnering with community members Conflict of Interest Statement The authors declare that the research was conducted in the absence of any commercial or financial relationships that could be construed as a potential conflict of interest. Acknowledge past injustices
Time commitment/Lack of perceived benefit/inadequate compensation	Incentives (clarify they are signs of respect/thanks)
Lack of awareness of trials/Assumptions about patient suitability	Marketing promotion Improved training materials
Communication barriers	Ask questions to reduce unmet concerns Simple words and open dialogue, underscoring that participation is completely voluntary (5 W’s)
Transportation	Arrange transportation
Lack of minority representation among research staff and physicians	Increase diversity within study team
Lack of culturally appropriate information	Culturally appropriate recruitment approaches in patient’s native language
Lack of access to care/recruitment at clinics	Decentralized trials

### Provider-level solutions

At the provider-level, physician enthusiasm and improved communication skills may improve the physician-patient relationship and increase recruitment numbers (70, 74, 76, 83). Simple questions such as “Is there something else you want to address in the visit today?” have been shown to reduce patient’s unmet concerns, ensuring that they are well-informed regarding the clinical trial (96). Further, lack of awareness of clinical trials among physicians can be lessened by marketing promotion and improving training and educational materials for physicians and other providers. Increasing the availability of information about the trials, as well as improving the quality of training materials will better prepare providers to understand eligibility requirements and will remind them of the existence of the project, increasing the likelihood of recruiting racial and ethnic minority individuals. Additionally, full transparency between providers/researchers and potential research participants can improve understanding of what it means to participate in research and mitigate fears surrounding the research process. The provider/researcher should use simple words to clearly explain the purpose of the research study, what will be asked of the participant, what will be done with the information, and emphasize the voluntary nature of participation. This method allows for open dialogue about participation in the project, while simultaneously underscoring that the patient is in control when choosing to participate and can opt to leave the study at any point (70, 75, 76, 83, 92, 94, 95). Furthermore, having a solid framework in place when having these conversations, such as “The 5 Ws of Racial Equity in Research Framework,” which encourages asking “Who, Why, When, What, and Where,” could provide a great starting point for providers and researchers to think about how they are going to have these conversations with potential racial and ethnic minority participants, to best anticipate their questions and come to a solution that will alleviate their concerns and allow them to gain the most from their participation in the study (97).

### Sponsor/system-level solutions

At the sponsor/system-level, in terms of transportation issues, one possible strategy to alleviate the burden would be to offer to arrange transportation services to and from the clinic for follow-up appointments, which may make research trials more accessible to those without access to a stable form of transit (76, 92). The costs of the transportation to and from the appointments is something that should be thought of ahead of time by researchers and built into the budget, rather than as an afterthought. In order to increase cultural sensitivity and navigate language barriers, physicians should enact culturally appropriate approaches to recruitment in the patients’ native language, removing a significant obstacle for patients and allowing them to fully understand the implications of participating in the trial. These approaches include training staff and researchers in cultural competence, designing language-concordant pamphlets and educational materials sensitive to patient cultural values, and making recruitment techniques more culturally sensitive (e.g., taking faith, religious, and cultural considerations into account) (76, 92, 93). As far as the large discrepancy in the number of URM providers/researchers and the effect this has on lack of minority recruitment, this is more difficult to address. Evidence suggests that emphasizing shared cultural attributes improves recruitment (90). However, a short-term solution could be to increase the diversity within the study team to include research assistants and coordinators that reflect the diversity of the local or national population. Additionally, decentralized trials present a solution for the recruitment difficulties faced by recruiting directly from clinics. Through the use of telehealth, social media marketing, remote patient monitoring devices, and other technologies, sponsors may be able to recruit and maintain participant involvement in trials through more convenient and cost-effective avenues, which require less time and money on behalf of the participant (98). Additionally, electronic medical records provide crucial demographic and medical information that can potentially be used to enroll and match members of minority groups in clinical trials. However, it is important to note that decentralizing trials may exclude people without adequate and consistent access to technology, which unfortunately tends to disproportionately affect racial and ethnic minority individuals (99). Notably, the utility of decentralized trials has gained national recognition with the recent approval of the Food and Drug Omnibus Reform Act (FDORA) of 2022, which requires the FDA to issue guidance for decentralized studies in an effort to increase diversity in clinical trial recruitment across the board. With these incoming FDA guidelines for the utilization of decentralized studies, it is important to target this expanding method of recruitment towards increasing trial participant diversity (100).

## Conclusion

Since improved control of T2D is imperative in preventing the development and progression of CVD, CKD, and ultimately death, strategies must be implemented to target disproportionately affected groups (such as Hispanic/Latino, non-Hispanic Black, and Asian individuals) and increase their representation in clinical trials. The CVOTs have demonstrated outstanding CV and kidney benefits for many of the novel drugs, specifically the SGLT2-inhibitors and GLP-1 RAs, yet the low percentage of racial and ethnic minority individuals included in the trials is indicative of the barriers that marginalized individuals face. Unsurprisingly, recent data indicate that Hispanic/Latinos and non-Hispanic Black individuals are prescribed these novel agents at lower rates than their non-Hispanic White counterparts (101, 102). While the reasons for this lack of prescribing has not been fully elucidated, it can be assumed that many of the barriers that contribute to the lack of recruitment of racial and ethnic minority individuals into clinical trials may also affect the implementation of medications in a real-world setting.

**Figure 2:**
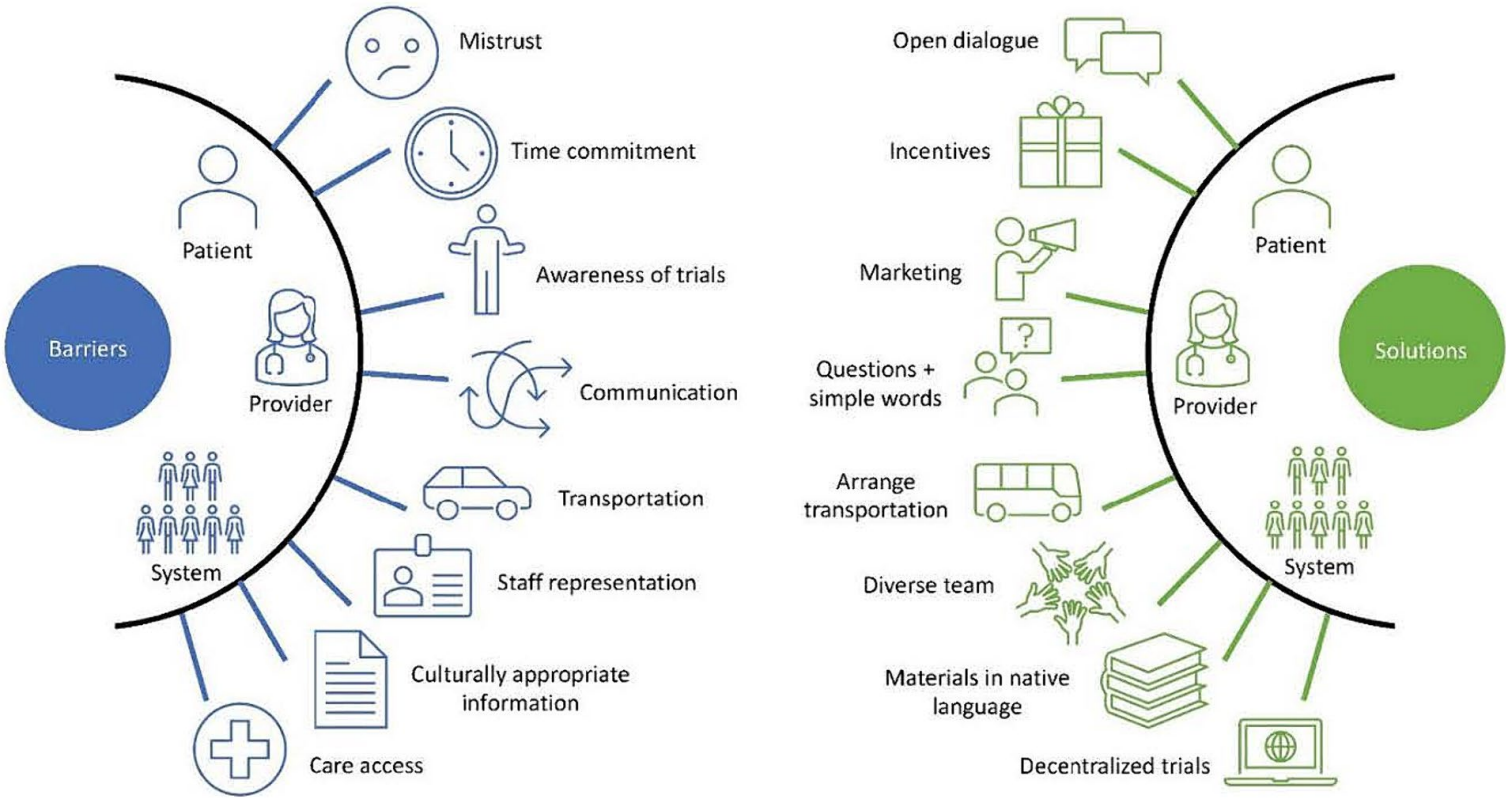
Barriers and solutions to improve clinical trial diversity.

The lack of involvement of racial and ethnic minority individuals in clinical trials is deeply problematic. Individuals disproportionately burdened by T2D and its complications need to be accurately represented in clinical trials if healthcare inequities are to be improved. Furthermore, the inclusion of these individuals in future trials will provide valuable information with regards to reasons for discontinuation, side effects, and overall efficacy of medications that may or may not be unique to these patient populations. The collection of this type of data would be incredibly valuable when it comes time for clinicians to discuss these drugs with their minority patients and make a shared decision regarding initiation. While many barriers and several valuable strategies to improve recruitment have been well-described in the literature, it is essential that researchers and clinicians alike adopt these strategies and conceptualize new ones to improve diversity in future clinical trials (Figure 2). It is also important to note that while the focus of this manuscript was on the CVOTs, the barriers noted and suggested strategies are equally important in improving minority recruitment in other types of clinical trials, including those that focus on patients without diabetes. Although recruiting diverse populations to participate in research is challenging, with proper planning and resource allocation, accomplishing this goal is possible and necessary to improve care and outcomes among racial and ethnic minority individuals. Finally, we acknowledge that while the recruitment of minorities into clinical trials will not completely address the health inequities faced and experienced by minorities, it represents one step, of many, that can help us to reduce inequities in health care.
